# Race and Other Disparate Demographic Variables Identified Among Emergency Department Boarders

**DOI:** 10.5811/westjem.2022.5.55703

**Published:** 2022-08-28

**Authors:** Robert C. Ruffo, Erin F. Shufflebarger, James S. Booth, Lauren A. Walter

**Affiliations:** University of Alabama at Birmingham Heersink School of Medicine, Department of Emergency Medicine, Birmingham, Alabama

## Abstract

**Introduction:**

Emergency department (ED) boarding, the process of holding patients in the ED due to a lack of inpatient beds after the decision is made to admit, has profound consequences. Increased ED boarding times are associated with adverse patient outcomes, including increased mortality. While previous studies have demonstrated racial disparities with regard to ED boarding, current literature lacks insight into discrepancies that may exist among other demographic groups as it pertains to ED boarding. We sought to review ED boarding times differentiated by demographic characteristics.

**Methods:**

We conducted a retrospective review of all ED admissions from an academic ED in the Southeast from April–September 2019. The primary outcome assessed was boarding time, defined as time from decision to admit to ED departure. Patient demographic data including race, gender, and age were collected and analyzed. We performed descriptive statistics and chi-square analyses.

**Results:**

The study population included 17,606 patients with a mean age of 56.3. Nearly half (49.8%) of the patients were female. Additionally, 43.8% of patients were Black and 48.6% White. For all admissions, there was no difference in mean boarding time among Black and White patients (5.2 ± 8.8 vs 5.2 ± 8.2 hours, *P* = 0.11). Among Emergency Severity Index (ESI) level I admissions, Black patients boarded longer than White patients (4.1 ± 0.3 vs 2.7 ± 0.3 hours, *P* = 0.009). Black patients also boarded significantly longer than White patients for psychiatric admissions (22.7 ± 23.7 vs 18.5 ± 19.4 hours, *P* <0.05). For all admissions, males boarded longer than females (5.5 ± 8.5 vs 4.9 ± 8.2 hours, *P* <.0001). Patients older than 75 boarded for less time (3.8 ± 6.2 hours) compared to younger groups (15–24: 6.4 ± 10.8 hours; 25–44: 6.6 ± 10.8; 45–64: 5.0 ± 7.6; and 64–75: 4.7 ± 6.7; all *P* <.05).

**Conclusion:**

This analysis demonstrated significant differences in ED boarding times between races among psychiatric and ESI I admissions, gender, and age. This data provides insight into differences in ED boarding times among demographic groups and provides a focal point for examining possible factors contributing to the observed differences.

## INTRODUCTION

Emergency department (ED) boarding, the process of holding patients in the ED due to a lack of inpatient beds after the decision is made to admit, is prevalent across hospitals throughout the United States (US). As of 2015, US inpatient beds have decreased by nearly one-third compared to 1975 while ED visits have significantly increased.^1^–^2^ Moreover, ED boarding time has been shown to be an important indicator of patient-centered outcomes. There is evidence that as ED boarding times increase, mortality and hospital length of stay (LOS) also increase in a nearly linear fashion.^3^ The medical literature has suggested numerous factors that likely contribute to adverse outcomes among boarding ED patients including delays in medication delivery, completion of orders, and nursing staff shortages.^4^–^6^

In 2009, Pines et al found that Black patients had significantly longer ED boarding times compared to non-Black patients in a large multicenter study that included over 14,000 patients.^7^ This study is relatively unique in that it clearly identified a disparity among a large sample of ED boarding patients. While this evidence is important, it was published over a decade ago with little additional research contributing to the topic of racial disparities and ED boarding in the interim. Because Black Americans suffer disproportionately from health disparities, it is vital that additional research be conducted to reveal more insight into potential underlying disparities in ED boarding across racial groups.

Moreover, it has been shown that ED boarding and psychiatric visit times are longer when compared to non-psychiatric ED encounters.^8^–^9^ According to 2016 data, nearly 10 million inpatient admissions across the US were associated with a psychiatric or substance use disorder and cost hospitals nearly $15.3 billion.^10^ Psychiatric patients are a vulnerable population due to tendencies of socioeconomic instability, high rates of concomitant substance misuse, and inconsistent access to healthcare resources. Because ED psychiatric visits are common, costly, and involve a susceptible population, it is crucial that disparities, if present, be identified to reduce potential adverse events and improve overall quality of care for this group.

Because there is a clear association with ED LOS and poor patient outcomes, it is important to identify factors associated with longer boarding times. While the medical literature does provide clear examples of disparities among Black and psychiatric patients, there is little recent literature regarding additional differences in ED boarding times across other demographic groups such as gender and age. We aimed to fill in these gaps in the literature and provide additional data on known disparities by identifying differences in ED boarding time across several demographic groups awaiting hospital inpatient beds in a large academic hospital in the Southeast.

## METHODS

This study is a retrospective review and analysis of all admissions from the University of Alabama at Birmingham (UAB)’s two hospital EDs over a six-month period from April–September 2019. This study, including data collection and analysis, has been reviewed and approved by the UAB Institutional Review Board. UAB is an urban, academic, tertiary care center. UAB ED averages approximately 73,000 patient visits annually. An additional site, UAB-Highlands-Highlands, located nearby on UAB’s southern campus, is a Level 1 geriatric ED and averages approximately 32,000 patient visits annually. Data analysis and statistical review began in December 2020.

**Population Health Research Capsule**

What do we already know about this issue?

*Black patients board significantly longer than White patients when admitted to hospitals through the ED. Longer boarding times are linked to increased mortality.*

What was the research question?

*Do other disparities exist among ED boarders across various demographic groups?*

What was the major finding of the study?

*We found longer ED boarding times for Black critically ill patients and psychiatric admissions, compared to Whites, and for all men and non-elderly patients.*

How does this improve population health?

*Identifying disparities among ED boarders may provide insight into underlying factors, and inform future studies.*

All patients seen at UAB ED are given an Emergency Severity Index (ESI) score ranging from I (most urgent) to V (least urgent) and have demographic data including gender, age, and race recorded in an electronic health record (EHR). The ESI is a triage tool integrated into the EHR for stratifying patients based on acuity and projected resource needs.^11^ Given the size of the hospital site with 1,207 inpatient beds, a central patient flow and bed control system is used for inpatient bed assignment. Once the decision is made to admit, the clinician places a bed request order in the EHR, which alerts the patient flow staff that the patient needs an inpatient bed assignment. The ED has little control over the patient’s ultimate destination aside from determining the level of care required in conjunction with the accepting inpatient team. Medical patients are assigned beds based on availability which varies depending on level of care required (acute, intermediate or intensive), hospital capacity and staff availability.

Our institution does have specialty intensive care units (ICU) (eg, cardiac, neurological, medical and trauma, surgical), but depending on resource availability, ED patients can be placed in the ICU that is the best fit. The patient flow staff may use age when determining bed assignment, as some units and services have certain age criteria. Other demographic factors are not immediately accessible during this process and are not typically reviewed. A different process is in place for psychiatric admissions, as the center for psychiatric medicine handles the bed assignments internally.

The primary outcome assessed in this study was boarding time, which we defined as time from decision to admit to ED departure. Using data stored in UAB’s EHR, we examined boarding time among various demographic categories such as race, gender, and age among psychiatric and medical admissions during the specified period. Efforts to limit bias were made by using secure datasets stored in the EHR. The study size consisted of all admitted patients during the specified time period at UAB’s two hospital EDs. We conducted the analysis using descriptive statistics and bivariate analysis with independent *t*-test and ANOVA. The statistical analysis was performed using JMP Pro 16 (JMP Statistical Discovery LLC, Cary, NC).

## RESULTS

During the study period, 17,606 patients were admitted; and we collected and analyzed demographic information and acuity level for mean boarding times as shown in Table 1. Missing demographic data ranged from 5.3% (gender, age) to 7.8% (race). Of the admitted patients, approximately half were male (50.2%) with a mean age of 56.3 ± 18.2 years. There were slightly more White patients (48.6%) than Black (43.8%). The vast majority (95.7%) of the patients admitted had an ESI score of II or III. The overall data for each demographic group stratified by ESI level is shown in Table 2.

When evaluating boarding time for all admissions by racial group, we found no significant difference in mean boarding time among White, Black, and other racial groups. Black patients boarded for a mean duration of 5.2 ± 8.8 hours, White patients for a mean of 5.2 hours ± 8.2 hours, and other racial groups boarded for a mean of 4.7 ± 6.6 hours (*P* = .111, F = 2.2.). However, the data also showed that among the sickest patients admitted to the hospital (ESI level I admissions), Black patients boarded significantly longer than White patients with a mean duration of 4.1 ± 0.3 hours compared to 2.7 ± 0.3 hours (*P* = 0.009).

While there was no significant difference in boarding time among racial groups for all admissions regardless of acuity, when examining admissions by particular type of admission, we found a significant difference in mean boarding time among Black and White psychiatric patients. Black patients (n = 401) awaiting psychiatric admission boarded for a mean duration of 22.7 ± 23.7 hours compared to White psychiatric patients (n = 526) who boarded for a mean duration of 18.5 hours ±19.4 (*P* = 0.0078). All other racial groups (n = 57) boarded for a mean duration of 17.8 ± 13.4 hours awaiting psychiatric admission as shown in Table 3.

Regarding male and female patients, there was a significant difference in mean boarding time. For all admissions, male patients boarded for a mean duration of 5.5 ± 8.5 hours while female patients boarded for a mean duration of 4.9 ± 8.2 hours [t = 4.32, dF = 16,665, *P* < .0001]. Additionally, among ESI level III patients, males boarded significantly longer than females for a mean duration of 4.9 ± 0.1 hours compared to 4.2 ± 0.1 hours (*P* < .0001). There were no additional differences between male and female patients based on acuity level or admission type.

Lastly, the data for ED boarding time for all admissions among age groups showed that patients in the ≥75 age group boarded for a significantly shorter duration than all other age groups with a mean duration of 3.8 ± 6.2 hours [ANOVA, F = 43.9, *P* < .001]. Additionally, patients ≤44 years had significantly longer boarding times than all other older age groups (*P* <.0001). There were no significant differences between age groups among psychiatric admissions. However, there were significant differences observed among age groups based on acuity level. For all ESI level II admissions, boarding times by age group were almost uniformly shorter as age increased. The ≥75 age group boarded for a mean of 4.1 ± 0.1 hours, which was significantly shorter compared to all other age groups (*P* <.0001). Additionally, boarding times were significantly shorter for the 65–74 age group (*P* <.0001) compared to the younger 25–44 and 15–24 age groups and boarding duration for the 45–64 age group was significantly shorter compared to respective younger age groups (*P* <.0001). For ESI level III admissions, boarding time for the ≥75 age group was significantly shorter compared to all younger age groups (*P* <.001) and the 45–64 age group boarded for significantly less time compared to the 25–44 age group (*P* <0.05). There were no significant differences among age groups for ESI level I, IV and V admissions.

## DISCUSSION

In this review we identified several significant trends with regard to demographic characteristics and ED boarding times. While Pines et al found significant differences in ED boarding time among racial groups for medical admissions in a large, multicenter study, our findings did not show significant differences across racial groups for all admissions; however, we did find significant differences in ESI I and psychiatric admissions. Generally, patients with an ESI I have life-threatening conditions and require immediate interventions and ultimately ICU admission. Because of this, ESI I admissions should have similar boarding times due to a shared need for critical resources, including rapid transportation to an inpatient unit. While the cause of this discrepancy is unclear, it demonstrates an obvious disparity in this subgroup. Because these are the sickest patients in the hospital, identifying underlying factors for this discrepancy in the future may have a profound impact on patient outcomes.

Existing literature suggests that patients admitted to psychiatric services have longer ED boarding times compared to patients admitted to medical services.^8^–^9^ However, none of these studies specifically examined differences in ED boarding times among racial groups in the psychiatric populations. Because we found that Black psychiatric admits board significantly longer than their White counterparts, we believe this to be a relatively novel finding that would be useful for future research. Psychiatric patients present with a variety of complaints ranging from mild depression to severe psychosis. Many patients with severe, acute mental illness (ie, psychosis and violent behavior) require more resources and higher security rooms for detention and monitoring than others. Because there may be limited high-security areas, psychiatric patients may experience longer boarding times than others. We did not examine differences in specific psychiatric diagnoses among racial groups; thus, it is possible that this may have contributed to the observed differences. Moreover, there is evidence that suggests that psychiatric boarding times may be related to individual insurance status.^12^ While it is possible that socioeconomic factors (insurance, access to transportation, etc.) play a large role in the overall care of psychiatric patients, it is unclear whether there are other underlying factors responsible for the observed differences that we found. Because of this, we believe that it is crucial for this vulnerable patient population to be studied further in the future.

Regarding gender, our findings showed that male patients had significantly longer ED LOS among general admissions and ESI III admissions. Generally, higher acuity patients are prioritized for available inpatient beds over less sick patients. The discrepancy for general admissions doesn’t appear to be related to acuity level as male patients had a higher overall total and relative proportion of higher acuity visits (ESI I and II) compared to females. Females were significantly older than males (57.4 ± 18.9 vs 55.2 ± 17.3 years, respectively). Because older patients typically board for less time compared to younger patients, it is possible that age affected the comparison between genders. However, there may be additional reasons for this discrepancy in our population that aren’t clear.

Additionally, our analysis of the age groups found that elderly patients boarded for significantly less time than the younger age groups. Our data revealed that the oldest patients boarded for the shortest mean duration for general admissions and among ESI II and III admissions. Interestingly, mean boarding time among the ESI II and III subgroups largely decreases as age increases. This finding suggests that the discrepancies in boarding time among age groups may be related to factors that are intrinsically more common among elderly age groups such as baseline health comorbidities, lower functional mobility, and age-related cognitive dysfunction. The literature is scant on the topic of ED boarding time as it relates to age; however, one previous study found that older and sicker patients experienced longer boarding times compared to younger age groups.^13^ Explanations for this discrepancy are lacking. Reasons for this are unclear; however, the extremes of age (ie, youngest and oldest patients) are often prioritized for inpatient beds.

The flow of patients through an ED to an inpatient unit requires multiple steps and complex coordination of communication, technology and, ultimately, physical interactions for patients to arrive at their final destination. These processes are admittedly complex and require thorough analysis that is outside the scope of this study to fully understand how to improve efficiency from a patient flow standpoint. However, our findings show clear differences in ED LOS among various groups and suggest the possibility that inherent patient demographics may somehow be impacting overall boarding times, in addition to the multifaceted mechanisms responsible for patient flow.

## LIMITATIONS

The main limitation to our study is that it was a single-center, retrospective analysis. This single-center design could limit generalizability to other institutions. Selection bias was minimized by using a dataset of all admissions over a six-month period before the coronavirus 2019 pandemic. However, there was a subset of this dataset with missing demographic information, which could have led to selection bias. Additionally, there was potential for bias with ESI level designation. While this system does have objective parameters considering patient acuity and resource needs, the final level is ultimately determined by a triage nurse whose decision could be affected by the patient’s gender, age, race, or chief complaint. Finally, we did not adjust for additional confounders including admission diagnosis, which could have led to bias in the study design.

## CONCLUSION

We found significant differences in ED boarding times among racial groups for ESI I and psychiatric admissions, gender, and among various age groups. There is strong evidence demonstrating the detrimental impact of long ED boarding times on overall patient outcomes, highlighting the importance of uncovering additional factors that may be causing the observed differences. Because our findings have not been previously well described in the literature, this data is a useful addition that may serve as a focal point for examining underlying clinical and social factors that may be contributing to the observed differences.

## Figures and Tables

**Table 1:** Boarding times shown by demographic group and acuity level.

Variable	n (%)	Boarding in hours (Mean ± SD)
Gender		
Female	8,308 (49.8)	→4.9 ± 8.2
Male	8,364 (50.2)	→5.5 ± 8.5
Race		
Black	7,116 (43.8)	5.2 ± 8.8
White	7,886 (48.6)	5.2 ± 8.2
Other	1,231 (7.6)	4.7 ± 6.6
Age (years)		
15–24	691 (4.1)	6.4 ± 10.8
25–44	3,998 (24.0)	6.6 ± 10.8
45–64	6,279 (37.7)	5.0 ± 7.6
65–74	2,938 (17.6)	4.7 ± 6.7
75+	2,764 (16.6)	→3.8 ± 6.2
ESI Level		
I	671 (3.8)	2.9 ± 3.6
II	9,150 (52.1)	5.6 ± 9.2
III	7,693 (43.8)	4.5 ± 7.0
IV	50 (0.3)	3.6 ± 4.2
V	4 (0.02)	0.7 ± 0.4

*ESI*, Emergency Severity Index.

**Table 2:** Mean boarding times by Emergency Severity Index level.

Variable	Boarding time in hours, mean ± SD (n, %)				

	ESI I (n = 671)	ESI II (n = 9,150)	ESI III (n = 7,693)	ESI IV (n = 50)	ESV V (n = 4)
Gender					
Female	3.2 ± 0.3 (216, 43.1)	5.8 ± 0.2 (3910, 46.5)	→4.2 ± 0.1 (4144, 54.0)	4.0 ± 5.2 (16, 32)	0.5 ± 0.3 (2, 50)
Male	3.5 ± 0.2 (285, 56.9)	6.1 ± 0.1 (4507, 53.5)	→4.9 ± 0.1 (3529, 46.0)	3.4 ± 3.8 (34, 68)	0.9 ± 0.3 (2, 50)
Race					
Black	→4.1 ± 0.3 (217, 44.9)	6.0 ± 10.4 (3279, 40.0)	4.4 ± 0.1 (3574, 47.8)	3.0 ± 3.2 (24, 49.0)	0.7 ± 0.3 (3, 75.0)
White	→2.7 ± 0.3 (224, 46.4)	5.9 ± 9.1 (4315, 52.7)	4.5 ± 0.1 (3319, 44.4)	4.8 ± 5.1 (18, 36.7)	0.7 ± 0.5 (1, 25.0)
Other	4.1 ± 0.6 (42, 8.7)	5.2 ± 7.2 (602, 7.3)	4.3 ± 0.3 (579, 7.7)	3.2 ± 5.0 (7, 14.3)	-----
Age (years)					
15–24	2.1 ± 0.8 (25, 5.0)	8.0 ± 0.5 (354, 4.2)	5.1 ± 0.4 (304, 4.0)	1.9 ± 2.0 (5, 10.0)	-----
25–44	2.6 ± 0.4 (105, 21.0)	8.1 ± 0.2 (1988, 23.6)	5.1 ± 0.2 (1867, 2.4)	4.2 ± 5.3 (16, 32.0)	0.5 ± 0.1 (2, 50.0)
45–64	3.6 ± 0.3 (189,37.8)	5.6 ± 0.2 (3165, 37.6)	4.5 ± 0.1 (2898, 37.8)	3.5 ± 4.2 (22, 44.0)	1.2 ± 0.2 (1, 25.0)
65–74	4.0 ± 0.4 (105, 21.0)	4.9 ± 0.2 (1496, 17.8)	4.5 ± 0.2 (1331, 17.3)	5.0 ± 2.5 (5, 10.0)	0.5 ± 0.2 (1, 25.0)
75+	3.4 ± 0.4 (76, 15.2)	→4.1 ± 0.3 (1413, 16.8)	→3.4 ± 0.2 (1273, 16.6)	0.9 ± 0.6 (2, 4.0)	-----

* Horizontal dashed lines denotes that no data for this particular category.

*ESI*, Emergency Severity Index.

**Table 3:** Mean boarding time by admission type.

Variable	Boarding time in hours, mean ± SD (n, %)	

	Medical admissions (n = 16,541)	Psychiatric admissions (n = 1,065)
Gender		
Female	→3.9 ± 0.1 (7,807, 50.0)	20.3 ± 0.9 (501, 47.8)
Male	→4.5 ± 0.1 (7,816, 50.0)	19.4 ± 0.9 (548, 52.2)
Race		
Black	4.1 ± 0.1 (6,715, 44.0)	→22.7 ± 23.7 (401, 40.8)
White	4.3 ± 0.1 (7,362, 48.3)	→18.5 ± 19.4 (524, 53.4)
Other	4.1 ± 0.2 (1,174, 7.7)	17.8 ± 13.4 (57, 5.8)
Age (years)		
15–24	3.8 ± 5.3 (539, 3.5)	15.8 ± 1.7 (152, 14.5)
25–44	4.3 ± 5.7 (3,431, 21.96)	20.3 ± 0.9 (567, 54.1)
45–64	4.4 ± 5.5 (6,016, 38.5)	20.3 ± 1.3 (263, 25.1)
65–74	4.4 ± 5.7 (2,891, 18.5)	22.3 ± 3.0 (47,4.5)
75+	→3.6 ± 4.6 (2,745, 17.6)	28.1 ± 4.8 (19, 1.8)
ESI Level		
I	2.9 ± 3.6 (670, 4.1)	1.7 (1, 0.09)
II	4.3 ± 5.8 (8,382, 50.8)	20.1 ± 20.6 (768, 72.6)
III	3.9 ± 5.0 (7,413, 44.9)	19.5 ± 21.7 (280, 26.5)
IV	2.7 ± 3.2 (41, 0.3)	7.8 ± 5.6 (9, 0.9)
V	0.7 ± 0.4 (4, 0.02)	-----

* Horizontal dashed lines denotes that no data for this particular category.

*ESI*, Emergency Severity Index.
